# Cargo proteins in extracellular vesicles: potential for novel therapeutics in non-alcoholic steatohepatitis

**DOI:** 10.1186/s12951-021-01120-y

**Published:** 2021-11-17

**Authors:** Jimin Kim, Seul Ki Lee, Seon-Yeong Jeong, Hye Jin Cho, Joonghoon Park, Tae Min Kim, Soo Kim

**Affiliations:** 1 Brexogen Research Center, Brexogen Inc., Songpa-gu, Seoul, 05855 South Korea; 2 Graduate School of International Agricultural Technology, Seoul National University, Pyeongchang, Gangwon-do 25354 South Korea; 3 Institutes of Green-Bio Science and Technology, Seoul National University, Pyeongchang, Gangwon-do 25354 South Korea

**Keywords:** Non-alcoholic steatohepatitis, Induced mesenchymal stem cells, Extracellular vesicles, Steatosis, Inflammation, Regeneration

## Abstract

**Background:**

Extracellular vesicles (EVs) are recognized as novel cell-free therapeutics. Non-alcoholic steatohepatitis (NASH) remains a critical health problem. Herein, we show that EVs from pan peroxisome proliferator-activated receptor agonist-primed induced mesenchymal stem cell (pan PPAR-iMSC-EVs) has unique cargo protein signatures, and demonstrate its therapeutic function in NASH.

**Results:**

A unique protein signatures were identified in pan PPAR-iMSC-EVs against those from non-stimulated iMSC-EVs. NASH mice receiving pan PPAR-iMSC-EVs showed reduced steatotic changes and ameliorated ER stress and mitochondiral oxidative stress induced by inflammation. Moreover, pan PPAR-iMSC-EVs promoted liver regeneration via inhibiting apoptosis and enhancing proliferation.

**Conclusions:**

We conclude that our strategy for enriching unique cargo proteins in EVs may facilitate the development of novel therapeutic option for NASH.

**Graphical Abstract:**

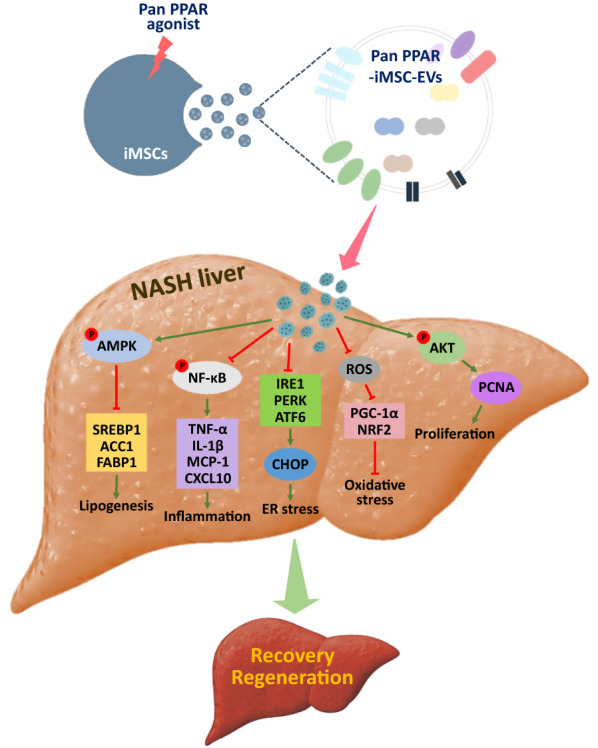

**Supplementary Information:**

The online version contains supplementary material available at 10.1186/s12951-021-01120-y.

## Background

Non-alcoholic fatty liver disease (NAFLD) is associated with metabolic disorders, including obesity, type 2 diabetes, and arteriosclerosis [1, 2]. NAFLD can develop into non-alcoholic steatohepatitis (NASH), an advanced form of fatty liver disease. The hallmarks of NASH include hepatic steatosis and inflammation, along with hepatocyte damage [2, 3]. Several underlying mechanisms, such as endoplasmic reticulum (ER) stress, oxidative stress, and inflammation, are responsible for the pathogenesis of NASH [4–7]. Characterized by steatosis, inflammation, ER stress, and parenchymal injury, NASH is an advanced and aggressive form of NAFLD which can progress to cirrhosis and hepatocellular cancer [8]. So far only a few drugs have shown early efficacy, and lifestyle modification remains the key to alleviating NASH/NAFLD [3].

Mesenchymal stem cells (MSCs) are multipotent stem/progenitor cells found in various adult tissues [9]. Despite their unique therapeutic potential [10], there are several limitations to the clinical applications of MSCs. In particular, they have limited proliferation capacity, and often undergo cellular senescence [11, 12]. Also, systemically administrated MSCs often accumulate in the lungs or liver [13, 14]. In vivo thrombogenesis or tumorigenesis is also an issue [15]. Extracellular vesicles (EVs) are nano-sized particles that play a critical role in intercellular communication by transporting specific biomolecular cargos, which are needed for maintaining tissue homeostasis [16–18]. Stem cell-derived EVs are reported to have potential to enhance recovery from tissue injury was confirmed in preclinical studies on cardiovascular, respiratory, skin, cartilage, renal diseases, and liver injuries [17, 19–23]. However, current protocols for preparing a sufficient number of EVs that can be experimentally or clinically used are challenging. Most importantly, prolonged culture period often leads to replicative senescence and the loss of differentiation potential. Also, cell surface marker for isolating large amount of homogenous MSCs is not available. Thus, the cellular characteristics often can differ among donors and isolation protocol [24–25]. In this regard, induced MSCs (iMSCs) formed from iPSCs is regarded as an alternative source for producing EVs because a large quantity of clonally-derived iMSCs can be generated in a scalable manner [27, 28], and their function in stimulating angiogenic, osteogenic, and cell survival pathways was demonstrated [30–32].

The therapeutic function of EVs can be enhanced by genetic modification or preconditioning strategy [34–36]. Recent studies demonstrated that the cargo protein in stem cell-derived EVs are associated with various pathways including cell proliferation, inflammation, metabolism, and tissue regeneration, demonstrating protein-based modes of actions of potentially therapeutic EVs [38–39]. Peroxisome proliferator-activated receptors (PPARs) belong to the family of ligand-activated nuclear receptors. Three PPARs, PPARα, PPARδ, and PPARγ, are expressed in tissue- or cell-specific manner, and they contribute to the improvement of glucose and lipid homeostasis, insulin resistance, and inflammation [41–43]. Importantly, pan PPAR agonist has shown effective outcomes in clinical trials on type 2 diabetes, NAFLD, and cutaneous systemic sclerosis [45–46], which prompted us to evaluate the potency of pan PPAR agonist as a priming factor for EV production.

Herein, we investigated whether EVs from pan PPAR agonist-stimulated iMSCs (pan PPAR-iMSC-EVs) can suppress NASH. We also identified protein sets enriched in pan PPAR-iMSC-EVs via mass spectrometry. To determine the therapeutic mechanisms, relevant in vitro studies were conducted.

## Results

### Characterization and biodistribution of pan PPAR-iMSC-EVs

**Fig. 1:**
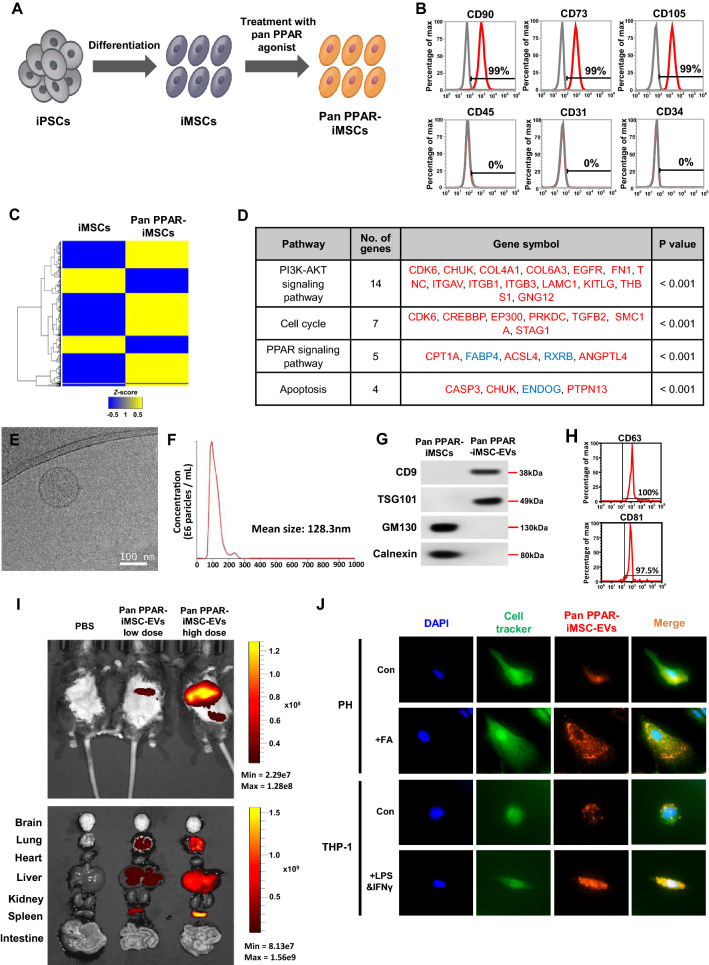
Characterization of pan PPAR-iMSCs and pan PPAR-iMSC-EVs. **A** Schematic diagram for the generation of induced mesenchymal stem cells (iMSCs) and pan PPAR-stimulated iMSCs (Pan PPAR-iMSCs). **B** Flow cytometric examination of markers positive (CD90, CD73, and CD105) or negative (CD45, CD31, and CD34) for pan PPAR-stimulated iMSCs. The IgG isotype was used as the control. **C** Representative heatmap for analysis of differentially expressed genes (DEGs) between iMSCs and pan PPAR-iMSCs. **D** Signaling pathways associated with pan PPAR-iMSCs. The upregulated and downregulated genes are depicted in red and blue, respectively. **E** Representative image of pan PPAR-iMSC-EVs observed using cryo-TEM. Scale bar = 100 nm. **F** Nanoparticle tracking analysis of pan PPAR-iMSC-EVs. **G** Immunoblot analysis of pan PPAR-iMSCs and pan PPAR-iMSC-EVs for markers of extracellular vesicles (CD9 and TSG101) or cellular organelles (GM130 and calnexin). **H** Flow cytometric analysis of pan PPAR-iMSC-EVs for CD63 and CD81. **I** In vivo tracking of pan PPAR-iMSC-EVs. The localization of fluorescently labeled pan PPAR-iMSC-EVs was visualized after 24 h of systemic administration. **J** Incorporation of DiD-labeled pan PPAR-iMSC-EVs in human primary hepatocytes (PH) treated without (first row) or with (second row) fatty acids for 24 h. THP-1 macrophages were treated with or without LPS and IFNγ (third and fourth row, respectively) for 24 h, and then the uptake of pan PPAR-iMSC-EVs was examined (600×magnification)

Figure 1A shows the schematic diagram of the generation of iMSCs and pan PPAR-stimulated iMSCs. Pan PPAR-stimulated iMSCs tested positive for MSC markers (CD90, CD73, and CD105) and negative for endothelial/hematopoietic markers (CD45, CD31, and CD34) (Fig. 1B). The protein expression of PPARα/γ/δ in iMSCs and pan PPAR-stimulated iMSCs was confirmed (Additional file 1: Fig. S1). Next, we analyzed the proteomic profile as well as the signaling pathways activated by the pan PPAR agonist in iMSCs. Treatment of pan PPAR agonist induced the upregulation of 335 genes and downregulation of 141 genes (Fig. 1C). Bioinformatic analyses showed that the signature of pan PPAR-stimulated iMSCs was significantly enriched in various pathways, including the PI3K-AKT, cell cycle, PPAR, and apoptosis signaling pathways (Fig. 1D), and the expression patterns were validated using qPCR (Additional file 1: Fig. S2). The average size of pan PPAR-iMSC-EVs was approximately 120–130 nm, as indicated in the cryo-transmission electron microscopy (cryo-TEM) and nanoparticle tracking analysis (NTA) analyses (Fig. 1E, F). Besides, western blot analysis revealed that pan PPAR-iMSC-EVs expressed the typical EV markers CD9 and TSG101 (Fig. 1G), whereas their expression was not observed in pan PPAR-stimulated iMSCs. Similarly, flow cytometric analysis showed that pan PPAR-iMSC-EVs tested positive for antibodies against CD63 and CD81, which are typical extracellular vesicles surface markers (Fig. 1H). In vivo tracking analysis revealed that pan PPAR-iMSC-EVs were present in several organs, including the spleen, liver, and lung, with an identifiable dose dependence (Fig. 1I). The incorporation of pan PPAR-iMSC-EVs was enhanced in primary hepatocytes or THP-1 macrophages, respectively, upon exposure to steatotic or inflammatory stimuli (Fig. 1J). Collectively, these results demonstrate that the pan PPAR agonist activated several intracellular pathways distinct from those in untreated cells, and pan PPAR-iMSC-EVs exhibited the typical characteristics of cell-derived extracellular vesicles, including the ability to incorporate into organs and cells.

### Signatures of pan PPAR-iMSC-EVs and connectivity map analyses

**Fig. 2:**
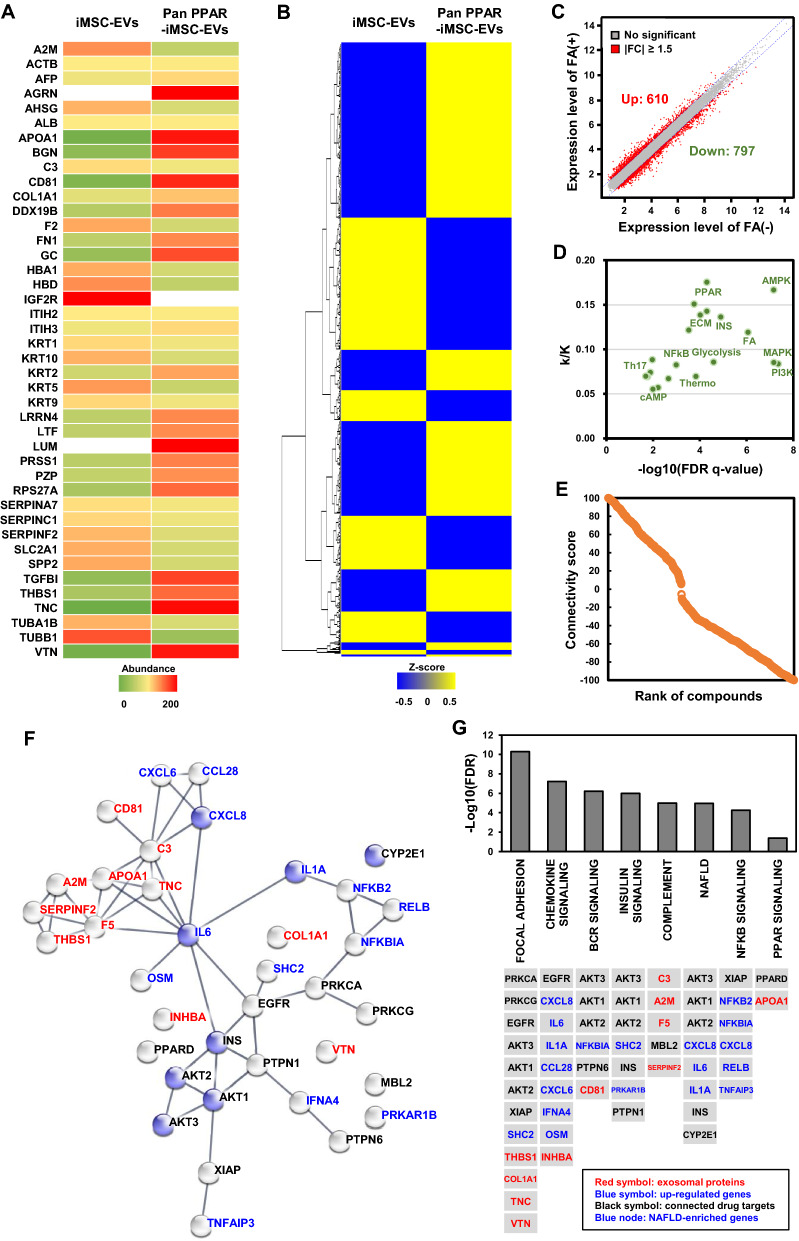
Signatures of pan PPAR-iMSC-EVs and pharmacological network analysis. **A** The proteomic signature of iMSC-EVs and pan PPAR-iMSC-EVs. The heatmap represents the abundance of EVs protein. **B** The transcriptomic signature of fatty acid-stimulated HepG2 cells that were subsequently treated with iMSC-EVs or pan PPAR-iMSC-EVs. The heatmap represents Z-score hierarchical clustering. **C** Scatter plot of DEGs in fatty acid-treated HepG2 cells. The vertical axis indicates the expression levels of genes in the vehicle-treated control, and the horizontal axis indicates those in fatty acid-treated HepG2 cells. **D** KEGG pathway enrichment of DEGs in fatty acid-treated HepG2 cells. The KEGG pathway represented by each dot is as follows. FA: fatty acid metabolism, INS: insulin signaling pathway, Thermo: thermogenesis, Th17: Th17 cell differentiation. The vertical axis indicates the q-value of each pathway, and the horizontal axis indicates the gene ratio. **E** The connected drug signature of pan PPAR-iMSC-EVs. The connected drugs are ranked according to the connective score with pan PPAR-iMSC-EVs. **F** The pharmacological network of pan PPAR-iMSC-EVs signature. The red nodes indicate the signature proteins of pan PPAR-iMSC-EVs, and the blue nodes indicate the NAFLD-enriched genes. The confidence level of the edge is more than 0.9. **G** KEGG pathway enrichment of the pan PPAR-iMSC-EVs signature in HepG2 cells. The enriched KEGG pathways are ranked using the (−) log-transformed q-value, and the genes identified in each pathway are depicted below. The red characters indicate the signature proteins of pan PPAR-iMSC-EVs, and the blue characters indicate the signature genes upregulated upon pan PPAR-iMSC-EVs treatment. The black characters indicate the targets associated with the signature drugs of pan PPAR-iMSC-EVs

Using LC–MS/MS, 18 proteins enriched in pan PPAR-iMSC-EVs were identified (|fold change|> 1.5, against iMSC-EVs), which included lumican (abundance = 200), tenascin (196.4), apolipoprotein A-1 (ApoA-1) (191.3), CD81 antigen (183.8), and thrombospondin-1 (155.7) (Fig. 2A, Additional file 2: Table S3). Subsequently, FA-stimulated HepG2 cells were treated with pan PPAR-iMSC-EVs, and the expression of 492 genes was found to be altered in pan PPAR-iMSC-EVs-treated cells compared to that in the untreated controls (288 and 204 upregulated and downregulated genes, respectively) (Fig. 2B, Additional file 2: Table S4). To understand the mechanism underlying hepatic steatosis, we analyzed the differentially expressed genes (DEGs) induced in response to FA treatment in hepatocytes. FA treatment induced the upregulation of 610 genes and downregulation of 797 genes compared to that in the vehicle control (Fig. 2C, Additional file 2: Table S5). No significant pathway with upregulated genes was detected. In contrast, downregulated genes were enriched in lipid metabolism- and inflammation-related pathways, including the PI3K-AKT signaling pathway (q = 4.388E−08), FA metabolism (q = 8.626E−07), insulin signaling pathway (q = 1.274E−05), PPAR signaling pathway (q = 4.999E−05), and Th17 cell differentiation (q = 2.016E−02) (Fig. 2D, Additional file 2: Table S6). To functionally predict the pharmacological outcome of pan PPAR-iMSC-EVs, we conducted a Connectivity map analysis. We found 18 drugs, including triciribine (AKT inhibitor, Connectivity score = 99.75), EI-273 (PKC inhibitor, 99.59), 4,5-dianilinophthalimide (EGFR inhibitor, 98.63), BRD-A94297859 (XIAP inhibitor, 98.49), and GW-0742 (PPAR receptor agonist, 94.6), associated with 59 target genes that were significantly similar to those expressed in the transcriptome profile of pan PPAR-iMSC-EVs (Fig. 2E, Additional file 2: Table S7). In addition, functional enrichment analysis revealed that 110 genes upregulated by pan PPAR-iMSC-EVs were significantly enriched in 117 canonical signaling pathways (q < 0.05). Therefore, signature of pan PPAR-iMSC-EVs was constructed using 108 genes identified through proteome, transcriptome, and Connectivity map analyses. The protein–protein interaction network and functional enrichment analyses revealed that signature of pan PPAR-iMSC-EVs was significantly enriched in various signaling pathways associated with lipid metabolism, fibrosis, and inflammatory responses, including focal adhesion (q = 5.7E−04), chemokine signaling pathway (q = 5.5E−03), NAFLD (q = 6.2E−03), NF-κB signaling pathway (q = 8.7E−03), insulin signaling pathway (q = 1.1E−02), and PPAR signaling pathway (q = 4.2E−02) (Fig. 2F, G, Additional file 2: Table S8), all of which are closely associated with metabolic diseases, including NASH [6]. The biochemical and pathway interaction data suggest the therapeutic potential of pan PPAR-iMSC-EVs in hepatic steatosis and inflammation.

### In vivo assessment of pan PPAR-iMSC-EVs function in the NASH model

**Fig. 3:**
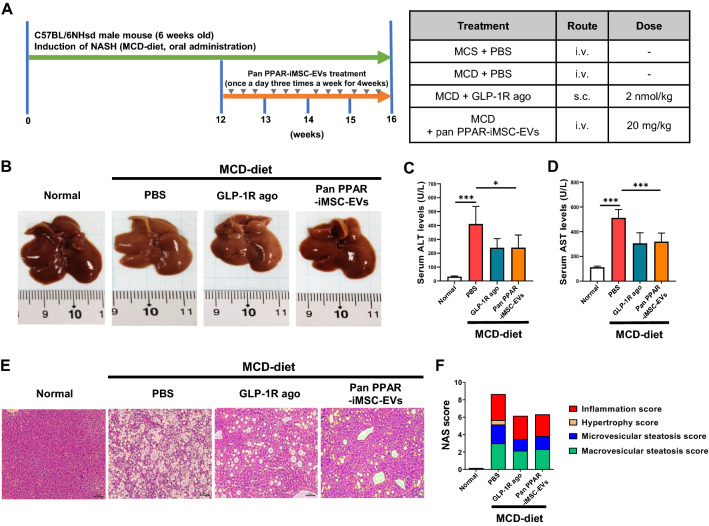
Effect of pan PPAR-iMSC-EVs on the restoration of hepatic structure and function. **A** Experimental scheme of NASH induction and pan PPAR-iMSC-EVs administration. **B** Representative images of liver tissues from normal mice and mice with MCD diet-induced NASH that received PBS or pan PPAR-iMSC-EVs. Mice treated with a GLP-1R agonist were used as the positive controls. **C, D** Serum ALT and AST levels in NASH mice that received PBS or pan PPAR-iMSC-EVs. Normal; n = 6, MCD-diet; n = 5. Data are presented as mean ± SD. *P < 0.05; ***P < 0.001. **E** Hematoxylin and eosin staining in liver tissues from mice administered an MCD diet and PBS or pan PPAR-iMSC-EVs. Scale bar: 50 μm. **F** Analysis of NAFLD activity score (NAS) using the index of inflammation, hypertrophy, and steatosis scores

The therapeutic function of pan PPAR-iMSC-EVs was examined using a mice model of NASH induced by MCD diet (Fig. 3A). The liver of mice fed the MCD diet turned pale compared to that of normal mice. In contrast, the liver of pan PPAR-iMSC-EVs-treated NASH mice was darker, similar to that observed in normal mice. In this model, used GLP-1 receptor agonist (Dulaglutide), a FDA-approved drug for type II diabetes as well as a prospective drug candidate for NAFLD or NASH [47, 48]. Contrary to expectation, no difference was observed in the livers from GLP-1R agonist- or PBS-treated mice (Fig. 3B). The whole liver weight did not differ between the PBS- and pan PPAR-iMSC-EVs-treated NASH mice (data not shown; MCD + PBS, 5.78 ± 0.12% vs. MCD + pan PPAR-iMSC-EVs, 5.33 ± 0.23%). Serum analysis revealed that the concentration of liver functional markers (alanine transaminase (ALT) and aspartate transaminase (AST)) decreased significantly in the MCD + pan PPAR-iMSC-EVs mice compared to that in the PBS-treated mice (Fig. 3C, D). In Fig. 3E, we found a decrease in lipid droplet accumulation and inflammatory cell infiltration in MCD + pan PPAR-iMSC-EVs mice compared to that in MCD + PBS mice. Detailed histological examination was performed by measuring the NAFLD activity score (NAS), which revealed that pan PPAR-iMSC-EVs reduced inflammation, hypertrophy, and steatosis (Fig. 3F). Collectively, these data indicate that pan PPAR-iMSC-EVs can restore the liver structure and function in NASH.

### Attenuation of hepatic steatosis by pan PPAR-iMSC-EVs

**Fig. 4:**
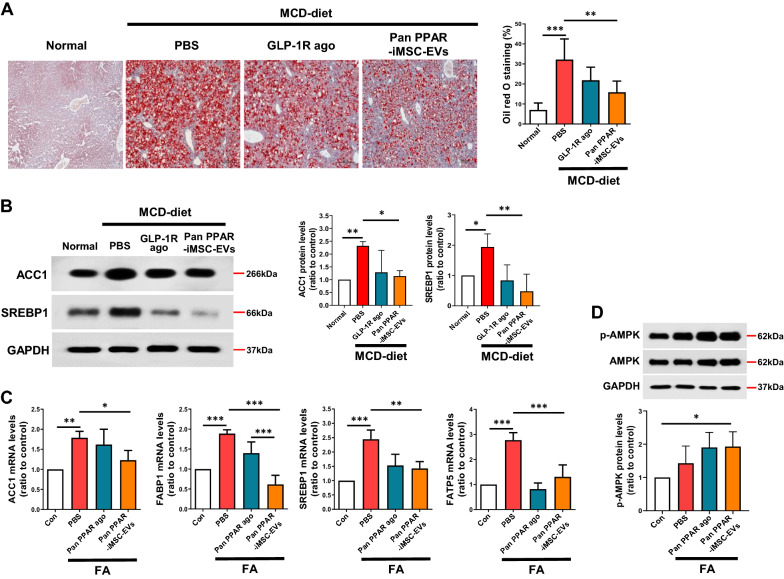
Attenuation of hepatic steatosis in mice with MCD diet-induced NASH by pan PPAR-iMSC-EVs. **A** Representative images of lipid droplet staining (Oil red O) in liver tissues from NASH mice and relative quantification. Scale bar: 200 μm. Normal; n = 6, MCD-diet; n = 5. Data are presented as mean ± SD. **P < 0.01; ***P < 0.001. **B** Analysis of ACC1 and SREBP1 protein expression in liver tissues from NASH mice. n = 4. Data are presented as mean ± SD. *P < 0.05; **P < 0.01. **C** qPCR analysis of lipogenic genes in primary hepatocytes after treatment with vehicle (PBS), pan PPAR agonist, or pan PPAR-iMSC-EVs. n = 4. Data are presented as mean ± SD. *P < 0.05; **P < 0.01; ***P < 0.001. **D** Immunoblot analysis of phospho-AMPK levels in fatty acid-stimulated primary hepatocytes after treatment with the vehicle (PBS), pan PPAR agonist, or pan PPAR-iMSC-EVs. The density of phosphorylated AMPK was normalized to that of total AMPK. n = 4. Data are presented as mean ± SD. *P < 0.05

In NASH, the dysregulation of lipid metabolism is associated with the upregulation of lipogenesis and reduction of very-low-density lipoprotein (VLDL) secretion [49]. Oil Red O staining revealed that lipid droplet deposition decreased in the liver tissue from the MCD + pan PPAR-iMSC-EVs mice (Fig. 4A). In addition, the expression of lipogenic proteins (ACC1 and SREPB1) was reduced in the liver tissue from MCD + pan PPAR-iMSC-EVs mice than in that from mice only fed an MCD diet (Fig. 4B). qPCR analysis of lipogenesis-related genes (ACC1, FABP1, SREBP1, and FATP5) in FA-stimulated primary hepatocytes revealed that their expression was reduced in the MCD + pan PPAR-iMSC-EVs group (Fig. 4C). We next analyzed the circulating free fatty acids (FFA) levels in blood from NASH mice, because excess FFAs secreted from adipose tissue are known to contribute to hepatic steatosis [50]. As shown in Additional file 1: Table S2, compared to that in mice only administered an MCD diet, the serum FFA levels were lower in MCD + pan PPAR-iMSC-EVs mice. Additionally, the serum VLDL levels in pan PPAR-iMSC-EVs-treated mice were higher than those in mice that only received an MCD diet, which is consistent with a previous study that accumulated hepatic TG is released into the circulation primarily as VLDL particles [51]. On the other hands, another contributing factor that causes hepatic TG accumulation in NASH is dysfunctional β-oxidation in the mitochondria. Unfortunately, we found that the mRNA expression of LCAD, CPT1α, and Acsl1 was not different in MCD + PBS and MCD + pan PPAR-iMSC-EVs mice (Additional file 1: Fig. S3). Finally, phospho-AMPK levels increased in FA-treated primary hepatocytes with pan PPAR-iMSC-EVs (Fig. 4D). Collectively, pan PPAR-iMSC-EVs improved hepatic steatosis by inhibiting lipogenesis and promoting VLDL release.

### Alleviation of inflammation by pan PPAR-iMSC-EVs

**Fig. 5:**
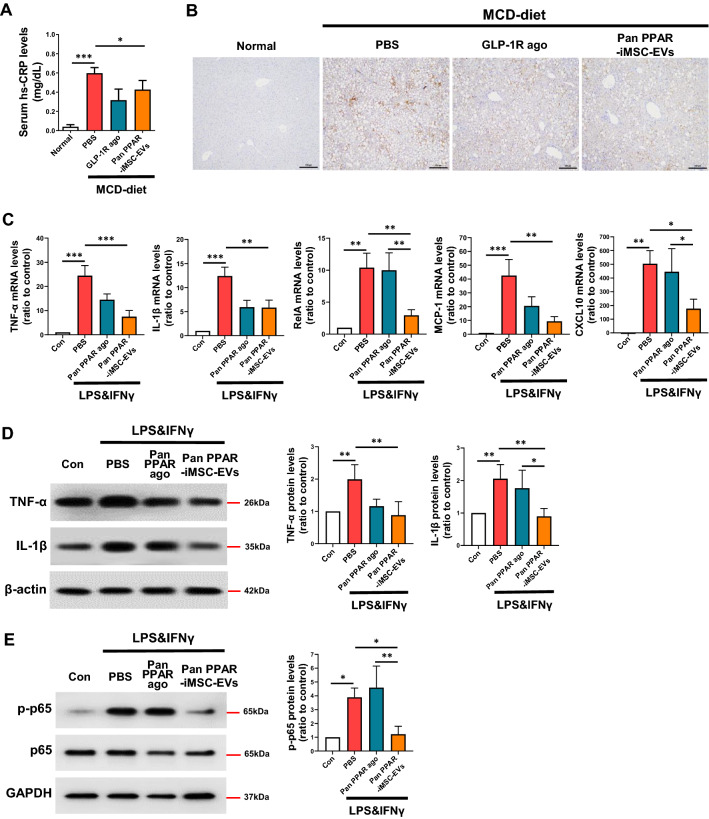
Attenuation of inflammation in MCD diet-induced NASH mice by pan PPAR-iMSC-EVs. **A** Serum level of hs-CRP in pan PPAR-iMSC-EVs-injected NASH mice. Normal; n = 6, MCD-diet; n = 5. Data are presented as mean ± SD. *P < 0.05; ***P < 0.001. **B** Immunohistochemical analysis of TNF-α protein in liver tissues. Scale bars: 100 μm. **C** mRNA expression analysis of inflammatory genes in LPS/IFNγ-stimulated THP-1 macrophages using qPCR. n = 3. Data are presented as mean ± SD. *P < 0.05; **P < 0.01; ***P < 0.001. **D** Immunoblot analysis of TNF-α and IL-1β in THP-1 macrophages treated with LPS/IFNγ in the presence of the vehicle (PBS), pan PPAR agonist, or pan PPAR-iMSC-EVs. n = 4. Data are presented as mean ± SD. *P < 0.05; **P < 0.01. **E** Immunoblot analysis of phosphorylated p65 (p-p65) expression in LPS/IFNγ-stimulated THP-1 in the presence of the vehicle (PBS), pan PPAR agonist, or pan PPAR-iMSC-EVs. The density of phosphorylated p65 was normalized to that of total p65. n = 3. Data are presented as mean ± SD. *P < 0.05; **P < 0.01

Persistent lipotoxicity caused by accumulation of harmful lipids on NASH leads to chronic hepatic inflammation [52]. We next evaluated the anti-inflammatory effects of pan PPAR-iMSC-EVs in NASH. A lower hs-CRP level was observed in mice treated with pan PPAR-iMSC-EVs compared to those treated with PBS (Fig. 5A). Consistently, the levels of hepatic TNF-α were low in pan PPAR-iMSC-EVs-treated NASH mice (Fig. 5B). Furthermore, pan PPAR-iMSC-EVs downregulated the mRNA expression of inflammatory genes (those encoding TNF-α, IL-1β, RelA, MCP-1, and CXCL10) in activated THP-1 macrophages (Fig. 5C). Immunoblot analysis also showed that the expression of TNF-α and IL-1β protein was reduced by pan PPAR-iMSC-EVs in activated THP-1 cells (Fig. 5D). Additionally, the activation of NF-κB signaling was suppressed by pan PPAR-iMSC-EVs in inflammatory THP-1 (Fig. 5E). These data support the claim that pan PPAR-iMSC-EVs alleviates NASH by playing an anti-inflammatory role.

### Improvement of ER and mitochondrial stresses by pan PPAR-iMSC-EVs

**Fig. 6:**
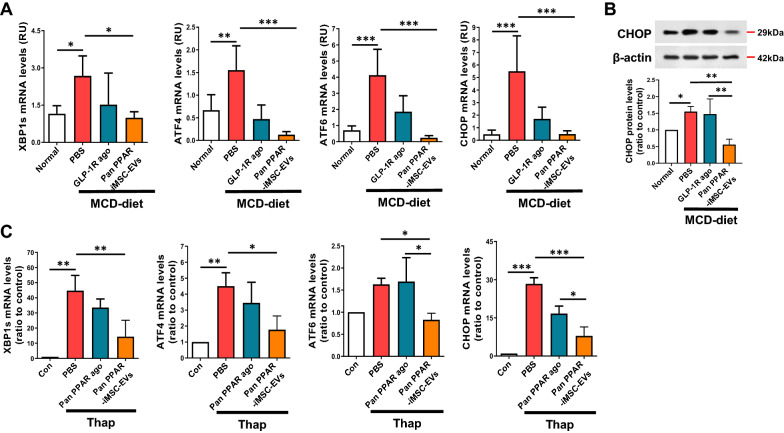
Pan PPAR-iMSC-EVs-mediated reduction of ER stress in hepatocytes and liver tissue. **A** Analysis of ER stress-responsive gene expression in the liver tissues of normal or NASH mice treated with vehicle (PBS), GLP-1R agonist, or pan PPAR-iMSC-EVs. Normal; n = 6, MCD-diet; n = 5. Data are presented as mean ± SD. *P < 0.05; **P < 0.01; ***P < 0.001. **B** Immunoblot analysis of CHOP in the liver tissues of NASH mice treated with vehicle (PBS), GLP-1R agonist, or pan PPAR-iMSC-EVs. n = 4. Data are presented as mean ± SD. *P < 0.05; **P < 0.01. **C** Analysis of ER stress-responsive gene expression in normal or thapsigargin-treated primary hepatocytes that were subsequently treated with the vehicle (PBS), pan PPAR agonist, or pan PPAR-iMSC-EVs. n = 3. Data are presented as mean ± SD. *P < 0.05; **P < 0.01; ***P < 0.001

**Fig. 7:**
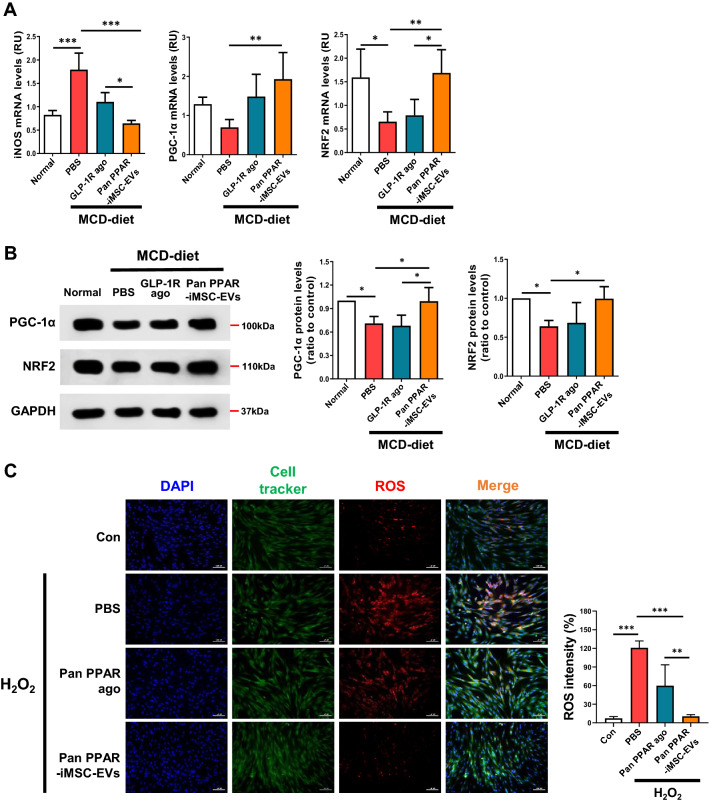
Pan PPAR-iMSC-EVs-mediated reduction of mitochondrial oxidative stress in hepatocytes and liver tissue. **A** qPCR analysis of genes involved in ROS generation (iNOS) and mitochondrial biogenesis (PGC-1α and NRF2) in the liver tissues of NASH mice treated with the vehicle (PBS), GLP-1R agonist, or pan PPAR-iMSC-EVs. Normal; n = 6, MCD-diet; n = 5. Data are presented as mean ± SD. *P < 0.05; **P < 0.01; ***P < 0.001. **B** Immunoblot analysis of PGC-1α and NRF2 in the liver tissues of NASH mice treated with PBS, GLP-1R agonist, or pan PPAR-iMSC-EVs. The bar charts are for the four replicates. n = 4. Data are presented as mean ± SD. *P < 0.05. **C** Comparison of ROS generation in primary hepatocytes treated with PBS, pan PPAR agonist, or pan PPAR-iMSC-EVs under oxidative stress. Scale bars: 100 μm. The intensity of ROS was normalized against that of nuclear staining. n = 4. Data are presented as mean ± SD. **P < 0.01; ***P < 0.001

NASH is metabolically associated with ER dysfunction [53]. We found that the mRNA expression of genes associated with ER stress-related pathways (XBP1s, ATF4, ATF6, and CHOP) was significantly suppressed in the liver of mice that received pan PPAR-iMSC-EVs (Fig. 6A). Consistently, the protein levels of CHOP, which is activated by XBP1, ATF4, and ATF6, were also reduced by pan PPAR-iMSC-EVs (Fig. 6B). Similarly, thapsigargin-induced ER stress was suppressed by pan PPAR-iMSC-EVs in human primary hepatocytes (Fig. 6C). Additionally, the expression of iNOS, an upstream stimulator of NOX2-mediated reactive oxygen species (ROS) generation, was reduced after pan PPAR-iMSC-EVs treatment. In contrast, the mRNA and protein expression of PGC-1α and NRF2, which are essential for mitochondrial biogenesis (thereby increasing lipid metabolism and decreasing ROS formation [54]), decreased in NASH mice, whereas it was maintained in pan PPAR-iMSC-EVs-treated mice (Fig. 7A, B). Next, primary hepatocytes were used to investigate whether ROS generation induced by oxidative stress could be suppressed by pan PPAR-iMSC-EVs. As a result, the pan PPAR agonist and pan PPAR-iMSC-EVs decreased the ROS levels in human primary hepatocytes, with the latter exhibiting better efficiency (Fig. 7C). Collectively, these data demonstrate that pan PPAR-iMSC-EVs plays homeostatic roles in enhancing cellular integrity by reducing ER stress, promoting mitochondrial biogenesis, and reducing ROS-mediated injury in hepatocytes.

### Regenerative potential of pan PPAR-iMSC-EVs in primary hepatocytes and liver tissue from NASH mice

**Fig. 8:**
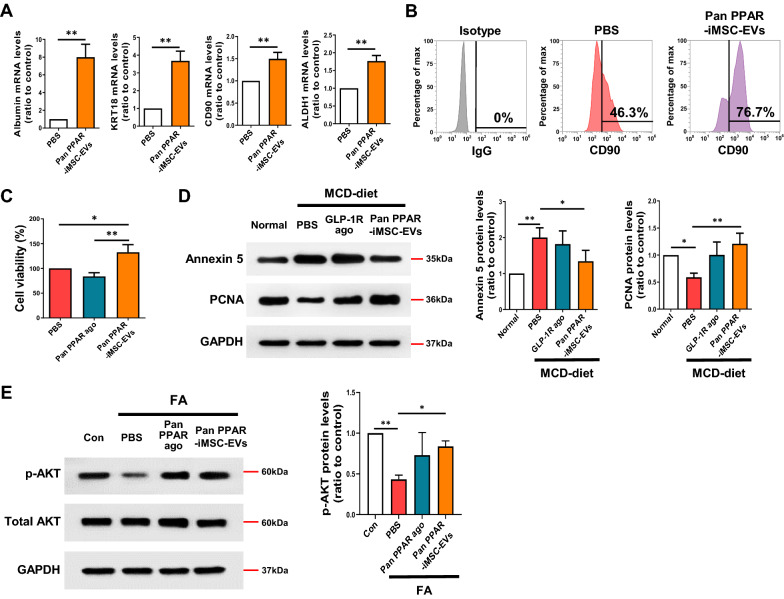
Regenerative potential of pan PPAR-iMSC-EVs in steatotic hepatocytes. **A** Expression analysis of genes indicative of maturity in primary hepatocytes after treatment with pan PPAR-iMSC-EVs. PBS was used in the control group. n = 4. Data are presented as mean ± SD. **P < 0.01. **B** Flow cytometric analysis of CD90 expression in primary hepatocytes after treatment with PBS or pan PPAR-iMSC-EVs. The isotype IgG control was used for comparison. **C** Comparison of the viability of primary hepatocytes after treatment with PBS, pan PPAR agonist, or pan PPAR-iMSC-EVs. n = 3. Data are presented as mean ± SD. *P < 0.05; **P < 0.01. **D** Immunoblot analysis of annexin5 and proliferating cell nuclear antigen (PCNA) in the liver tissues from NASH mice treated with PBS, GLP-1R agonist, or pan PPAR-iMSC-EVs. n = 4. Data are presented as mean ± SD. *P < 0.05; **P < 0.01. **E** Immunoblot analysis of AKT in PBS-, pan PPAR agonist- or pan PPAR-iMSC-EVs-pretreated human primary hepatocytes that were subsequently stimulated with fatty acids. The density of phosphorylated AKT was normalized to that of total AKT. n = 3. Data are presented as mean ± SD. *P < 0.05; **P < 0.01

The activation of progenitor cells and regeneration of parenchymal cells are vital to the recovery of injured organs [55]. qPCR analysis revealed that the Albumin and KRT18, which is representative of the mature hepatocytes [56], or CD90 and ALDH1, which is the marker of hepatic progenitor cells [57, 58], gene expressions were upregulated upon pan PPAR-iMSC-EVs treatment in human primary hepatocytes (Fig. 8A). Also, flow cytometric analysis showed that cells expressing CD90 were upregulated in pan PPAR-iMSC-EVs-treated human primary hepatocytes (Fig. 8B). Additionally, pan PPAR-iMSC-EVs increased the viability of primary hepatocytes (Fig. 8C). Immunoblot analysis using Annexin 5 and PCNA antibodies showed that pan PPAR-iMSC-EVs treatment decreased apoptotic cell death, whereas it promoted the proliferation of hepatocytes in the NASH liver (Fig. 8D). Lastly, AKT phosphorylation in FA-stimulated human primary hepatocytes increased upon pre-treatment with pan PPAR-iMSC-EVs (Fig. 8E). Altogether, these results suggest that pan PPAR-iMSC-EVs can potentially block NASH progression by reviving the hepatocytes and also by enhancing cell survival.

## Discussion

Our data show that pan PPAR-iMSC-EVs ameliorated the progression of NASH as shown by the gross morphology and histological analysis of liver tissue, serum liver function markers, NAS score, and the reduced lipid droplet deposition in the liver of NASH mice. Consistently, in vitro studies with primary hepatocytes and activated THP-1 monocytes corroborated the anti-lipogenic and anti-inflammatory functions of pan PPAR-iMSC-EVs. With respect to cellular homeostasis, pan PPAR-iMSC-EVs reduced the ER stress, stimulated mitochondrial biogenesis, while reduced ROS generation in NASH liver of mice and human primary hepatocytes. In addition, pan PPAR-iMSC-EVs augmented the expression of genes associated with the mature or progenitor stages of human primary hepatocytes. In addition, pan PPAR-iMSC-EVs stimulated the PI3K-AKT pathway in steatotic human primary hepatocytes, and a higher number of proliferating hepatic cells were observed in pan PPAR-iMSC-EVs-treated NASH mice. Thus, we provide evidence for the therapeutic role of pan PPAR-iMSC-EVs in NASH via its anti-steatotic, anti-inflammatory, and tissue-regenerative function.

Recently, MSCs have been shown to exert therapeutic functions in experimental acute and chronic liver disease models, and several clinical studies on the function of stem cells in NASH is currently underway [21, 59]. EVs of various stem cells can alleviate liver inflammation and fibrosis by reducing oxidative injury, regulating inflammation, and stimulating proliferation of parenchymal cells, representing a novel therapeutic strategy for various liver injuries [59, 60]. Further, recent studies have demonstrated that MSC-EVs improves liver fibrosis, and that enhance liver regeneration [61]. Although the therapeutic role of naïve MSC-EVs have been confirmed in numerous disease animal models, their therapeutic outcome can significantly vary [16]. Also, the procedures for maintaining the potential of naïve MSC-EVs is needs to be further optimized [62]. Thus, various attempts were made to enhance the therapeutic potential of MSCs by priming approaches using cytokines, growth factors, drugs, hypoxic culture, genetic modification, or biomaterials [63].

PPAR has been one of the most active therapeutic targets for NASH during last years, and their subtypes including PPARα, PPARδ and PPARɣ has been reported to play homeostatic role in the liver [46]. Lanifibranor, a pan PPAR agonist, is currently under phase 3 clinical trial [64], demonstrating its minimal safety concern as well as its well-defined mode of action. Notably, it was recently documented that lanifibranor plays anti-steatotic and anti-inflammatory role in liver fibrosis, NASH, and mild NAFLD [44, 65]. Given these facts, we used lanifibranor for stimulating iMSCs, and found that pan PPAR-iMSCs were enriched with multiple pathways including PI3K-AKT signaling, PPAR, cell cycle and apoptosis regulation (Fig. 1D).

In NASH, dysregulation of lipid metabolism is associated with the upregulation of lipogenesis and reduced VLDL secretion [49, 66]. Our data demonstrate the anti-steatotic function of pan PPAR-iMSC-EVs in NASH, as evidenced by improved histological morphology, decreased micro/macro-vesicular steatosis, and reduced lipid deposits in liver from pan PPAR-iMSC-EVs mice (Fig. 3). The potential of MSC-derived EVs in reducing steatosis was also supported by a previous study; HFD-fed obese mice showed markedly ameliorated hepatic steatosis via reduction of liver weight, macrovesicular steatosis, and hepatic TG levels by EVs from adipose-derived stem cells [67]. Moreover, hepatic TG levels and the expression of lipogenic genes and proteins was increased in NASH liver, while decreased upon treated with pan PPAR-iMSC-EV (Fig. 4A–C). Emerging evidence have shown that activation of AMPK, an energy-sensing enzyme, is critical for improving metabolic syndrome such as NAFLD and type 2 diabetes [68, 69]. Similarly, we showed that hepatic steatosis was reduced by pan PPAR-iMSC-EVs via regulation of AMPK activation (Fig. 4D). Serum VLDL levels were increased in pan PPAR-iMSC-EVs mice compared with VLDL levels in those that received an MCD diet only (Additional file 1: Table S2), which is consistent with previous study [66]. We also demonstrated that pan PPAR-iMSC-EVs ameliorated hepatic inflammation by blocking p65 phosphorylation (Fig. 5). This finding is consistent with a previous study that demonstrated the anti-inflammatory function of EVs from amnion-derived MSCs (AmMSC-EV); AmMSC-EV suppressed the expression of LPS-stimulated inflammatory cytokines and chemokines in HFD-induced NASH rats as well as in Kupffer cells via inhibiting p65 phosphorylation and resulting NF-κB transcriptional activity [70].

NASH undergoes a series of events that lead to ER stress [71], which is caused by unfolded protein response (UPR) [53] and ROS generation [72]. In addition, ER stress induces expression of CHOP, a transcription factor that mediates apoptotic cell death [73]. It is important to note that CHOP can not only induces apoptosis but also can act as a key player in the pathophysiology of NASH by activating NF-kB and increasing TNF-a in hepatocytes [74], possibly contributing the overall improvement of NASH. We demonstrated that the expression of CHOP was reduced in NASH mice upon pan PPAR-iMSC-EVs administration, with a concomitant interruption of PERK, IRE1α, and ATF6, which are upstream inducers of CHOP (Fig. 6A–C). Thus, we argue that pan PPAR-iMSC-EVs potently reduced ER stress by suppressing CHOP activity.

Hepatic lipid overload induces the overproduction of oxidants by affecting several ROS-generating mechanisms [75], and ROS generated by alterations in mitochondrial function play a significant role in NASH [76]. We showed that pan PPAR-iMSC-EVs reduced ROS generation (as shown by increased NRF2/PGC-1α and decreased ROS activity; Fig. 7A, B). Our findings are consistent with other previous study that demonstrated the anti-oxidative function of exosome-rich fractionated sercretome in APAP or H_2_O_2_-induced liver cells [77]. Similarly, it was also previously shown that human umbilical cord MSC-EVs decreased ROS and mitochondrial superoxide levels in H_2_O_2_-exposed hepatocytes [78]. Pan PPAR-iMSC-EVs were also effective in promoting liver regeneration, as shown by increased proliferation and reduced apoptosis via activating PI3K-AKT pathway (Fig. 8). Collectively, we demonstrate that pan PPAR-iMSC-EVs improve NASH microenvironments by reducing ER stress, mitochondrial oxidative stress, and apoptosis.

## Conclusions

In conclusion, we demonstrated that EVs from pan PPAR-iMSCs has anti-steatotic, anti-inflammatory, and tissue-repairing function, contributing to a marked improvement of NASH. Our data may contribute to developing a biologically-active and innovative cell-free nanotherapeutics.

## Materials and methods

### Animals

Six-week-old C57BL/6 male wilvvd-type mice were obtained from Koatech Co., Ltd. (Korea) and fed either chow diet (n = 6) or an MCD diet (n = 5) for 12 weeks. Animal care and procedures were approved in the rodent animal facility area of Knotus Co., Ltd. (Korea; Approval Number: 19-KE-265). At 18 weeks into the MCD diet, 2 nmol/kg dulaglutide (GLP-1 receptor agonist) was subcutaneously injected into mice every other day for 4 weeks, and 20 mg/kg pan PPAR-iMSC-EVs was intravenously injected once a day, three times a week, for 4 weeks. At the end of the experiments, the mice were anesthetized, and their serum and liver tissues were collected. The following environmental conditions were maintained: temperature, 23 ± 3 ℃; relative humidity, 55% ± 15%; ventilation, 10–20 air changes/h; luminous intensity, 150–300 Lux; and a 12 h light/12 h dark cycle.

### Cell culture

For cell maintenance, human primary hepatocytes (ScienCell, Carlsbad, CA, USA) were cultured in hepatocyte basal medium supplemented with 5% fetal bovine serum (FBS), 1% penicillin, and growth supplements (ScienCell). THP-1 monocytes (ATCC, Manassas, VA, USA) were cultured in Roswell Park Memorial Institute Medium (RPMI 1640; Gibco, Waltham, MA, USA) supplemented with 10% FBS (HyClone, Chicago, IL, USA) and 1% antibiotic–antimycotic solution (Thermo Fisher Scientific, Waltham, MA, USA). To establish the NASH in vitro model, human primary hepatocytes were treated with 100 mM FA (oleate-palmitate, 2:1 molar ratio) in Dulbecco’s modified Eagle’s medium (DMEM) supplemented with 2% FBS for 48 h and then treated with 100 μg/mL pan PPAR-iMSC-EVs with 100 mM FA in serum-free DMEM for 24 h. In addition, pan PPAR-iMSC-EVs was simultaneously treated with 500 nM thapsigargin in serum-free DMEM for 24 h. However, the THP-1 monocytes were stimulated with 200 ng/mL phorbol-12-myristate-13-acetate (PMA), 100 ng/mL lipopolysaccharide (LPS), and 20 ng/mL IFNγ in RPMI 1640 medium supplemented with 10% FBS for 24 h. Subsequently, THP-1 monocytes were treated with 100 μg/mL pan PPAR-iMSC-EVs, 100 ng/mL LPS, and 20 ng/mL IFNγ in serum-free DMEM for 24 h. The cells from both cell lines were cultured at 37 °C under 5% CO_2_ and 95% humidified air. To assess the phosphorylation levels, human primary hepatocytes and THP-1 macrophages were treated with 100 μg/mL pan PPAR-iMSC-EVs for 24 h. Following this, 100 mM FA or 200 ng/mL PMA, 100 ng/mL LPS, and 20 ng/mL IFNγ were mixed with serum-free DMEM and added to primary hepatocytes for 30 min (phospho-AKT and phospho-AMPK) or to THP-1 macrophages for 10 min (phospho-p65).

### Culture and RNA-Seq analysis of pan PPAR agonist-stimulated iMSC

iMSC were prepared as described in our previous study [79]. iMSC (passage 4) cultured in high-glucose DMEM (HyClone) supplemented with 15% FBS and 1% antibiotic–antimycotic solution (Thermo Fisher Scientific) in a T-75 flask (Eppendorf, Hamburg, Germany) at 37 ℃ in 5% CO_2_ and 95% humidified air. Upon reaching 90% confluence, the cells were detached using TryPLE Express (Thermo Fisher Scientific) and seeded at a density of 10,000 cells/cm^2^ in a 4-layer Cell Factory System (Thermo Fisher Scientific). The next day, the cells were treated with 10 μM lanifibranor (Cayman, Ann Arbor, MI, USA) for 24 h, after which the media were aspirated, and the cells were washed with Dulbecco’s phosphate-buffered saline (DPBS) (HyClone). RNA sequencing was performed using the application provided by Macrogen Inc. Hierarchical clustering was analyzed using complete linkage and Euclidean distance as a measure of similarity to present the patterns of differentially expressed transcripts, which were satisfied with |fold change|≥ 2 and p < 0.05 (independent *t*-test). Gene set enrichment and pathway analyses for significant gene list were performed using g: Profiler (https://biit.cs.ut.ee/gprofiler/gost) and KEGG database (http://www.genome.jp/kegg/pathway.html).

### Isolation of pan PPAR-stimulated iMSC EVs

EV-depleted FBS was prepared as described previously [80]. Pan PPAR-stimulated iMSCs were replaced with phenol red-free DMEM (Gibco, Waltham, MA, USA) supplemented with 15% EV-depleted FBS. After 3 days of incubation, the culture medium was harvested, centrifuged for 10 min at 300×*g*, and the supernatant was centrifuged for 20 min at 2000×*g*. The supernatant was centrifuged for an additional 80 min at 10,000×*g*. Thereafter, the supernatant was filtered through a 0.2 μm vacuum filter (Merck Millipore, Burlington, MA, USA). Lastly, pan PPAR-iMSC-EVs were isolated by ultracentrifugation at 100,000×*g* for 80 min, and the pellet was subsequently washed with PBS and subjected to ultracentrifugation (Beckman Coulter, CA, USA). The pan PPAR-iMSC-EVs pellets were resuspended in PBS.

### Cryo-TEM

A 200-mesh copper grid (MiTeGen, Ithaca, NY, USA) coated with formvar/carbon film was subjected to hydrophilic treatment. The pan PPAR-iMSC-EVs suspension (4 μL) was placed on a grid and blotted for 1.5 min at 100% humidity and 4 ℃. The pan PPAR-iMSC-EVs on the grid were visualized at 36,000 × magnification using a Talos L120C FEI transmission electron microscope (Thermo Fisher Scientific) at 120 kV.

### NTA assay

Measurements of particle size distribution and concentration of pan PPAR-iMSC-EVs were performed using a NanoSight NS300 instrument (Malvern Panalytical, Malvern, UK) based on NTA. For the analysis, pan PPAR-iMSC-EVs were diluted in sterile PBS (1:100) to reach the optimal volume for NTA. Measurements were performed at room temperature ranging from 23.0 to 25.2 °C using a 488 nm Blue laser and an sCMOS camera in several repeats. Sample analysis was conducted for 10 min under the following camera settings and processing conditions: Shutter 600, Gain 250, camera level 10, NTA version 3.0 0064, and Detection Threshold 10.

### Labeling of pan PPAR-iMSC-EVs with DiR and DiD and fluorescent imaging

Pan PPAR-iMSC-EVs were incubated with 1 μg/mL DiR buffer for 10 min at 37 °C according to the protocol mentioned by Lipophilic Tracers (Invitrogen, Waltham, MA, USA). Next, the DiR-labeled pan PPAR-iMSC-EVs were centrifuged for 80 min at 100,000×*g* and 4 °C and washed with PBS (Gibco). Lastly, 200 or 400 μg of DiR-labeled pan PPAR-iMSC-EVs was resuspended in 0.1 mL of PBS and intravenously injected into C56BL/6 mice. At 24 h, DiR-labeled pan PPAR-iMSC-EVs were detected using an In Vivo Imaging System (Caliper Life Sciences, Waltham, MA, USA) at excitation and emission wavelengths of 740 and 790 nm, respectively. The intensity of the region of interest was plotted in units of the maximum number of photons per second per centimeter square per steradian (p/s/cm^2^/sr). The procedure for preparing DiD-labeled pan PPAR-iMSC-EVs was identical to the procedure described above. DiD-labeled pan PPAR-iMSC-EVs were used to treat the human primary hepatocytes or THP-1 macrophages for 24 h with or without each stimulus. At 24 h, DiD-labeled pan PPAR-iMSC-EVs were observed under a Nikon Eclipse Ti2-U fluorescent microscope (Nikon, Tokyo, Japan).

### Bioinformatic analyses

After treatment of HepG2 cells with 100 mM FAs (oleate-palmitate, molar ratio 2:1) for 6 h, total RNA was isolated using the RNeasy Mini Kit (Qiagen, Hilden, Germany). The recovered RNA was profiled using the GeneChip® Human Gene 2.0 ST array (Affymetrix, Santa Clara, CA, USA). The fold change cutoff for FA-induced DEGs was set at 1.5. The DEGs were subjected to Gene Set Enrichment Analysis with KEGG collection at an FDR q-value cutoff of 0.05 (http://www.gsea-msigdb.org/gsea). The signature of pan PPAR-iMSC-EVs was constructed through transcriptomic, proteomic, and Connectivity map analyses. Briefly, FA-treated HepG2 cells were treated with pan PPAR-iMSC-EVs, and total RNA was profiled as described above. The DEGs induced by pan PPAR-iMSC-EVs was identified at a fold change cutoff of 1.5. Proteins enriched in pan PPAR-iMSC-EVs were qualitatively and quantitatively identified using LC–MS/MS (ProteomeTech Inc., Seoul, Korea). The DEGs induced by pan PPAR-iMSC-EVs was subjected to Connectivity map analysis, and drugs with a similar transcriptome profile as that of pan PPAR-iMSC-EVs and their target genes were identified. The DEGs induced by pan PPAR-iMSC-EVs, proteins of pan PPAR-iMSC-EVs, and target genes of pan PPAR-iMSC-EVs-like drugs identified were confirmed as pan PPAR-iMSC-EVs signatures. The established signature of pan PPAR-iMSC-EVs was subjected to protein–protein interaction network and functional enrichment analyses with interaction confidence of 0.9 (https://string-db.org).

### Flow cytometry

Pan PPAR-iMSC-EVs were stained using human MACSPlex Exosome Kit (Miltenyi Biotec, Bergisch Gladbach, Germany), and analyzed using an Attune NxT flow cytometer (Thermo Fisher Scientific). For analyzing the effect of pan PPAR-iMSC-EVs on hepatocyte regeneration, the hepatocytes were stained with anti-human CD90 APC-Cy7 antibody (BioLegend, San Diego, CA, USA) after pan PPAR-iMSC-EVs treatment and analyzed using the Attune NxT flow cytometer (Thermo Fisher Scientific). To confirm whether pan PPAR agonist-stimulated iMSCs express the typical cell surface markers for MSCs, pan PPAR agonist-stimulated iMSCs were stained with CD73 APC, CD105 PE, CD45 FITC, CD31 PE, and CD34 APC (eBioscience, Waltham, MA, USA) and CD90 APC-Cy7 (BioLegend) antibodies. Flow cytometric analysis was conducted using an Attune NxT flow cytometer (Thermo Fisher Scientific).

### Serum biochemical examination

Serum samples were collected 4 weeks after the initiation of pan PPAR-iMSC-EVs injection, and the levels of the following molecules were measured using a blood biochemical analyzer (7180, Hitachi, Japan): ALT, AST, TG, glucose, total cholesterol, high-density lipoprotein cholesterol, low-density lipoprotein cholesterol, lactate dehydrogenase, and gamma-glutamyltransferase.

### Real-time qPCR

Total RNA was isolated from liver tissues and various cell types using TRIzol® (Ambion, Waltham, MA, USA). cDNA was synthesized using 1 μg of total RNA using AccuPower® CycleScript RT PreMix dT_20_ (Bioneer, Daejeon, South Korea). Amplification was performed using the PowerSYBR® Green PCR Master Mix (Applied Biosystems) according to the manufacturer’s protocol. The gene expression levels were analyzed using real-time qPCR with the QuantStudio™ 5 Real-Time RCR System (Applied Biosystems). The primer sequences are listed in Additional file 1: Table S1. GAPDH was used as the reference for normalizing the differences in the quantity of mRNA in each sample. The relative gene expression levels were analyzed using the comparative 2^−ΔΔCt^ method. Each experiment was performed in triplicate.

### Western blot analysis

Cells or liver tissues were lysed in NP40 (Life Technologies, Carlsbad, CA, USA) or RIPA lysis buffer (Thermo Fisher Scientific) supplemented with protease inhibitors (Thermo Fisher Scientific). The protein concentration was measured using the Bradford Assay™ Reagent (Thermo Fisher Scientific) according to the manufacturer’s protocol. Samples were diluted at a 3:1 ratio using the 4 × Laemmli buffer (Bio-Rad Laboratories, Hercules, CA, USA) and heated at 100 °C for 10 min. Proteins were loaded and separated on precast polyacrylamide Mini-PROTEAN TGX gels (Bio-Rad Laboratories) and transferred to PVDF membranes (Bio-Rad Laboratories). The membranes were blocked with EveryBlot Blocking Buffer (Bio-Rad Laboratories) for 5 min and then treated overnight with primary antibodies at 4 °C. All primary antibodies were diluted in the EveryBlot Blocking Buffer. Antibodies against GM130, PCNA, AMPK, phospho-AMPK (Thr172), phospho-p65 (Ser536), pan AKT, phospho-AKT (Thr308) (Cell Signaling Technology, Leiden, The Netherlands), CD9, calnexin, IL-1β, p65, annexin5, β-actin, GAPDH (Abcam, Cambridge, UK), anti-TSG101, CD81 (Invitrogen), TNF-α, PGC-1α, NRF2, and CHOP (Novusbio, Centennial, CO, USA) were used as the primary antibodies. Western blotting for all target proteins, except CD81, was performed under reducing conditions. The membranes were washed for 10 min for five times and then treated with the secondary antibodies for 1 h. Anti-rabbit IgG and anti-mouse IgG (Abcam) antibodies were used as the secondary antibodies. After the membranes were washed for 10 min for five times, the target proteins were detected using the ECL Select™ Western Blotting Detection Reagent (GE Healthcare, Little Chalfont, UK) and analyzed using the ChemiDoc Imaging System (Bio-Rad Laboratories).

### ELISA and FFA assay

ELISA and FFA assay kits were performed using commercially available mouse ELISA kits. ELISA kits for insulin (Novusbio), hs-CRP (R&D systems), and VLDL (MyBioSource, San Diego, CA, USA) were used according to the manufacturer’s instructions. The assay sensitivity was < 0.19 ng/mL for insulin, < 0.015 ng/mL for hs-CRP, and < 0.195 ng/mL for VLDL. The intra- and inter-assay coefficients of variance were < 5.9% and < 6.4% for insulin, < 7.7% and < 10.8% for hs-CRP, and < 8% and < 10% for VLDL, respectively. Quantification of FFA was performed using commercially assay kit (Abcam), according to the manufacturer’s instructions.

### Histopathological analysis

Liver tissues were fixed in 10% paraformaldehyde and subjected to general tissue treatment, such as cutting, dehydration, and paraffin embedding. The tissues were sectioned at 5 μm and mounted on the slides. The specimens were deparaffinized using xylene. The tissues were rehydrated and stained with hematoxylin & eosin (H&E). In case of Oil red O staining, the tissues were embedded using OCT compound (Sakura Finetek, Torrance, CA, USA) and then sectioned at 20 μm using a cryotome (Leica, Wetzlar, Germany). The histopathological samples obtained were analyzed using Zen 2.3 blue edition image analyzer (Carl Zeiss, Oberkochen, Germany), and the values were normalized as the percentage of the staining area by total area. NAS examination was performed according to the histological criteria, and the levels of macrovesicular steatosis, microvesicular steatosis, and hypertrophy were scored from 0 to 3, based on the observation of the occupied area by the total area. Both steatosis and hypertrophy were evaluated at magnifications of 40× to 100× . For inflammation score, five fields were selected randomly and provided scores from 0 to 3.

### Immunohistochemical staining

Glass slides with slices of liver tissue were placed in a drier maintained at 60 °C, dried for 1 h, and deparaffinized using xylene. The tissues were rehydrated and incubated with 0.03% peroxidase for 15 min to block endogenous peroxidase activity. Antigen retrieval was performed by incubating the tissue sections with Tris–EDTA buffer (pH 9.0) at 121 °C for 15 min using a pressure cooker. To prevent non-specific reactions, 4% BSA and dextran was added for 30 min. Subsequently, the sections were incubated with the anti-TNF-α (Abcam) primary antibody for l h and then incubated with the anti-rat IgG H&L (Abcam) secondary antibody for 30 min at room temperature under gentle agitation. Samples were subsequently visualized under a BX53 biological microscope (Olympus, Tokyo, Japan), and representative images were captured for analysis.

### ROS assay

Primary hepatocytes were stimulated with 400 μM H_2_O_2_ for 24 h. Subsequently, H_2_O_2_-stimulated primary hepatocytes were treated with pan PPAR-iMSC-EVs in serum-free DMEM culture media for 24 h, followed by washing with DPBS. Briefly, CellROX® Reagent (Life Technologies) was mixed in serum-free DMEM at a final concentration of 5 μM and added to the human primary hepatocyte culture. The human primary hepatocytes were incubated for 30 min at 37 °C. Following staining, the cells were fixed in 4% paraformaldehyde (Fujifilm Wako Chemicals, Richmond, VA, USA) for 10 min and then washed three times with DPBS. The nuclei and cell bodies were counterstained with NucBlue™ Fixed Cell stain or CellTracker™ (Life Technologies), respectively. After this process, all samples were observed using Nikon Eclipse Ti2-U (Nikon, Tokyo, Japan), and the percentage of ROS-positive cells was analyzed based on nuclei intensity.

### Cell viability assay

Primary human hepatocytes (2 × 10^3^ cells/mL) were seeded in 96-well plates and cultured for 4 h in serum-free DMEM at 37 ℃ and 5% CO_2_. The absorbance (OD value) was measured at 450 nm using a multiplate reader (Thermo Fisher Scientific). The effects of pan PPAR-iMSC-EVs on the viability of primary hepatocytes were evaluated using the Cell Counting Kit-8 (Enzo life sciences, Farmingdale, NY, USA) according to the manufacturer’s instructions.

### Statistical analyses

Statistical analyses were performed using SPSS (version 18.0 for IBM, Chicago, IL, USA). For comparisons involving three or more groups, one-way analysis of variance (ANOVA) was used followed by Tukey’s post hoc test. For comparisons involving only two groups, the paired Student’s t-test was used. Data are expressed as means ± standard deviation (SD), and values with P < 0.05 were considered statistically significant.

## Supplementary Information

**Additional file 1: Fig. S1.** Protein expression of PPARs in iMSCs. PPARα/γ/δprotein expression in iMSCs treated with pan PPAR agonist. Human adipocyte is used as positive control. **Fig. S2.** Validation of gene expression in iMSCs and pan PPAR-iMSCs.Data are represented as mean ± SD. n = 4.*P < 0.05; **P < 0.01. **Fig. S3.** No effect of mitochondrial β-oxidation under treatment with pan PPAR-iMSC-EVs. mRNA expression of mitochondrial β-oxidation-related genes (LCAD, CPT1α, and Acsl1) in MCD-diet mouse injected pan PPAR-iMSC-EVs. Data are represented as the mean ± SD. Normal; n = 6, MCD-diet; n = 5.*P < 0.05; **P < 0.01 vs. Normal. **Table S1.** Sequences of primers used for real-time qPCR analysis. **Table S2.** Effects of the alteration of pan PPAR-iMSC-EVs treatment on serum metabolic parameters in MCD-diet mice at 4 weeks of treatment.

**Additional file 2: Table S3.** Proteomic analysis of exosomal proteins in iMSC-EV and pan PPAR-iMSC-EV. **Table S4.** Pan PPAR-iMSC-EV-induced differentially expressed genes in fatty acid-treated HepG2 cells. **Table S5.** Fatty acid-induced differentially expressed genes in HepG2 cells. **Table S6.** Representative KEGG pathways enriched with fatty acid-induced differentially expressed genes in HepG2 cells. **Table S7.** Connectivity map analysis of drugs and their target genes similar to that in the pan PPAR-iMSC-EV group. **Table S8.** KEGG enrichment analysis of pan PPAR-iMSC-EV signatures.

## Data Availability

All data generated or analyzed during this study are included in this published article.

## Abbreviations

ALT: Alanine transaminase
ApoA-1: Apolipoprotein A-1
AST: Aspartate transaminase
Cryo-TEM: Cryo-transmission electron microscopy
DEGs: Differentially expressed genes
DMEM: Dulbecco’s modified Eagle’s medium
DPBS: Dulbecco’s phosphate-buffered saline
ER: Endoplasmic reticulum
EVs: Extracellular vesicles
FFA: Free fatty acids
H&E: Hematoxylin & eosin
iMSCs: Induced mesenchymal stem cells
LPS: Lipopolysaccharide
MSCs: Mesenchymal stem cells
NAFLD: Non-alcoholic fatty liver disease
NAS: NAFLD activity score
NASH: Non-alcoholic steatohepatitis
NTA: Nanoparticle tracking analysis
Pan PPAR-iMSC-EVs: EVs from pan peroxisome proliferator-activated receptor agonist-primed induced mesenchymal stem cell
PMA: Phorbol-12-myristate-13-acetate
PPARs: Peroxisome proliferator-activated receptors
ROS: Reactive oxygen species
UPR: Unfolded protein response
VLDL: Very-low-density lipoprotein
