# Supplementary material for: Cargo proteins in extracellular vesicles: potential for novel therapeutics in non-alcoholic steatohepatitis
Source: J Nanobiotechnology. 2021 Nov 17;19:372. doi: 10.1186/s12951-021-01120-y (PMC8600817; doi:10.1186/s12951-021-01120-y)
Supplement: Supplementary file 1 — Additional file 1: Fig. S1. Protein expression of PPARs in iMSCs. PPARα/γ/δprotein expression in iMSCs treated with pan PPAR agonist. Human adipocyte is used as positive control. Fig. S2. Validation of gene expression in iMSCs and pan PPAR-iMSCs.Data are represented as mean ± SD. n = 4.*P < 0.05; **P < 0.01. Fig. S3. No effect of mitochondrial β-oxidation under treatment with pan PPAR-iMSC-EVs. mRNA expression of mitochondrial β-oxidation-related genes (LCAD, CPT1α, and Acsl1) in MCD-diet mouse injected pan PPAR-iMSC-EVs. Data are represented as the mean ± SD. Normal; n = 6, MCD-diet; n = 5.*P < 0.05; **P < 0.01 vs. Normal. Table S1. Sequences of primers used for real-time qPCR analysis. Table S2. Effects of the alteration of pan PPAR-iMSC-EVs treatment on serum metabolic parameters in MCD-diet mice at 4 weeks of treatment. [file 12951_2021_1120_MOESM1_ESM.pdf]

Additional file 1

Cargo proteins in extracellular vesicles: potential for novel therapeutics in non-alcoholic steatohepatitis

Jimin Kim[1], Seul Ki Lee[1], Seon-Yeong Jeong[1], Hye Jin Cho[2], Joonghoon Park[2,3], Tae Min Kim[2,3*], and Soo Kim[1*]

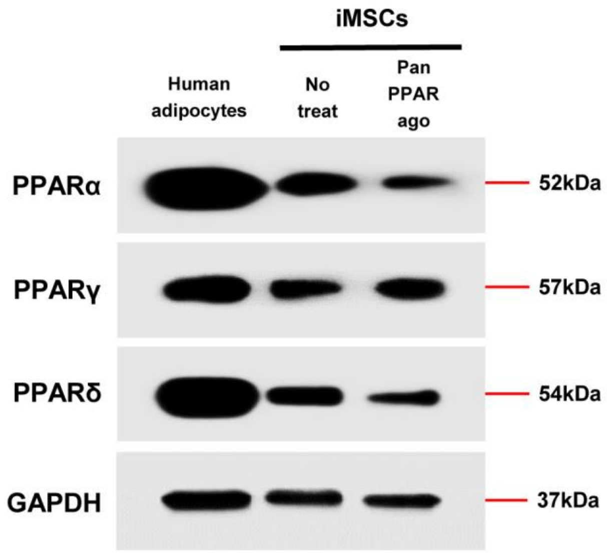

Fig. S1 Protein expression of PPARs in iMSCs. PPARα/γ/δ protein expression in iMSCs treated with pan PPAR agonist. Human adipocyte is used as positive control.

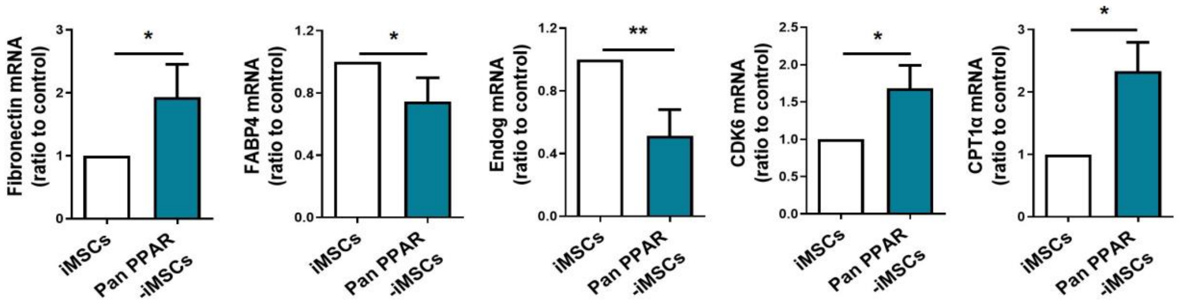

Fig. S2 Validation of gene expression in iMSCs and pan PPAR-iMSCs. Data are represented as mean ± SD. n = 4. *P < 0.05; **P < 0.01.

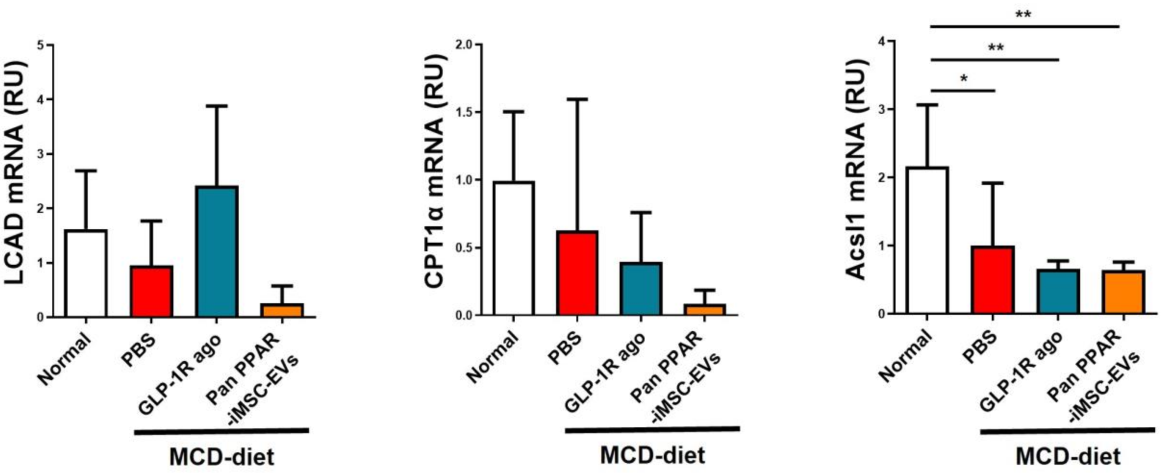

Fig. S3 No effect of mitochondrial β-oxidation under treatment with pan PPAR-iMSC-EVs. mRNA expression of mitochondrial β-oxidation-related genes (LCAD, CPT1α, and Acsl1) in MCD-diet mouse injected pan PPAR-iMSC-EVs. Data are represented as the mena ± SD. Normal; n = 6, MCD-diet; n = 5. *P < 0.05; **P < 0.01 vs. Normal.

Table S1. Sequences of primers used for real-time qPCR analysis.

Gene	Forward (5'–3')	Reverse (5'–3')
hGAPDH	ACATCGCTCAGACACCATG	TGTAGTTGAGGTCAATGAAG
mGAPDH	TGTCCGTCGTGGATCTGAC	CCTGCTTCACCACCTTCTTG
hFibronectin	AAGATTGGAGAGAAGTGGGA	GAGCAAATGGCACCGAGATA
hChuk	TGTAAAGGCCTGTGATGTTCCT	GGTTTGTTGAGCAGCTTTCGG
hEndog	AGTCGTACGTGCTGTGCTA	TCCTCGCGGAAGTCGCA
hCDK6	CTTGCTCCAGTCCAGCTACG	TGGGAGTCCAATCACGTCCA
hCPT1α	ACAGTCGGTGAGGCCTCTTATGAA	TCTTGCTGCCTGAATGTGAGTTGG
hACC1	GCTCCTTGTCACCTGCTTCT	CAAGGCCAAGCCATCCTGTA
hFABP1	GGGGAGAAAGTCAAGACAGTGG	TGGTGATTATGTCGCCGTTG
hSREBP1	CGGCGCTGCTGACCGACATC	CCCTGCCCCACTCCCAGCAT
hFATP5	TACAACACCGGGGACGTACT	CACCTTACCCTCACAACCTGG
mLCAD	CAAACGTCTGGACTCCGGTT	ACGTAAGCTTTTGCAATCGGG
mCPT1α	AGCTCGCACATTACAAGGACA	CCAGCACAAAGTTGCAGGAC
mAcsl1	AGGGTGAGGTGTGTGTGAAAG	CCAATGTCCCCCGTGTGTAA
hTNF-α	GAGCTGAACAATAGGCTGTTCCCA	AGAGGCTCAGCAATGAGTGACAGT
hIL-1β	ACAGCTGGAGAGTGTAGATCC	CTTGAGAGGTGCTGATGTACC
hRelA	GACCTGAATGCTGTGCGGC	ATCTTGAGCTCGGCAGTGTT
hMCP-1	TCTGTGCCTGCTGCTCATAG	GGGCATTGATTGCATCTGGC
hCXCL10	TGGCATTCAAGGAGTACCTCTC	TGATGGCCTTCGATTCTGGA
hXBP1s	ACTGCCAGAGATCGAAAGAAGG	CTAGCAAAAGTTTTTGGTTCTCTTC
hATF4	GGCCAAGCACTTCAAACCTC	GAGAAGGCATCCTCCTTGCT
hATF6	AATGTGTCTCCCCTTCTGCG	AGGCTGCCCTCTCAGAAAAC
hCHOP	AGGGAGAACCAGGAAACGGAAACA	TCCTGCTTGAGCCGTTCATTCTCT
mXBP1s	CGTGAGACTCGGTCTGGAAA	AACTGGGCCTTAGGGTGGAT
mATF4	CAGACACCGGCAAGGAGGAT	AGAGCTCATCTGGCATGGTT
mATF6	GTTGTCTAGTTCTCAGTCCCCC	GAGTCAGTCCATGTTCTGTTTT

		G
mCHOP	GGAAGCCTGGTATGAGGATCTG	GTGAAGGTTTTTGATTCTTCCTCTT
miNOS	GCGAAAGGTCATGGCTTCAC	CTGGTCCATGCAGACAACCT
mPGC-1α	TCAAGCCACTACAGACACCG	TAGCTGTCGTACCTGGGCCT
mNRF2	CCATTCCCGAATTACAGTGTCT	GACCACAGTTGCCCACTTCT
hAlbumin	TTCCAGGGGTGTGTTTCGTC	AAAGGCAATCAACACCAAGGC
hKRT-18	GAGGGCTCAGATCTTCGCAA	CCAGCTGCAGTCGTGTGATA
hCD90	CCTTCACTAGCAAGGACGAGG	TCACACTTGACCAGTTTGTCTCT
hALDH1	CCGTGGCGTACTATGGATGC	CGCAATGTTTTGATGCAGCCT

h, Human; m, Mouse.

Table S2. Effects of the alteration of pan PPAR-iMSC-EVs treatment on serum metabolic parameters in MCD-diet mice at 4 weeks of treatment

	MCD-diet		
	PBS	GLP-1R agonist	Pan PPAR -iMSC-EVs
n	5	5	5
Glucose (mg/dL)	53.2 ± 10.8	62.3 ± 12.7	88.0 ± 24.5*
Insulin (ng/mL)	26.7 ± 2.0	13.2 ± 4.0*	17.2 ± 5.3*
LDH (U/L)	1837.1 ± 532.3	1431.4 ± 371.9	1273.0 ± 205.8
GGT (U/L)	0.29 ± 0.26	0.22 ± 0.18	0.28 ± 0.27
FFA (μM)	36.1 ± 14.2	29.5 ± 10.3	25.7 ± 8.5
TC (mg/dL)	16.9 ± 3.8	28.2 ± 6.4*	33.8 ± 8.6*
LDL (mg/dL)	1.09 ± 0.19	2.60 ± 2.10	1.85 ± 0.36
VLDL (mg/dL)	0.51 ± 0.07	0.62 ± 0.13	1.43 ± 0.19*
HDL (mg/dL)	7.20 ± 0.97	13.4 ± 1.9*	14.5 ± 5.8*
ApoA-1 (ng/mL)	28.7 ± 12.9	55.4 ± 37.0	70.4 ± 14.2*

Data are shown as mean ± SD on the original scale. *P < 0.05 *vs.* PBS group analyzed using one-way ANOVA followed by Tukey's post hoc test.
